# *ERBB2* Amplification as a Predictive and Prognostic Biomarker in Upper Tract Urothelial Carcinoma

**DOI:** 10.3390/cancers15092414

**Published:** 2023-04-22

**Authors:** Annette Zimpfer, Said Kdimati, Melanie Mosig, Henrik Rudolf, Heike Zettl, Andreas Erbersdobler, Oliver W. Hakenberg, Matthias Maruschke, Björn Schneider

**Affiliations:** 1 Institute of Pathology, University Medical Center Rostock, 18057 Rostock, Germany; 2 Institute for Biostatistics and Informatics in Medicine and Ageing Research, University Medical Center Rostock, 18057 Rostock, Germany; 3 Clinical Cancer Registry, University of Rostock, 18055 Rostock, Germany; 4 Department of Urology, University Medical Center Rostock, 18057 Rostock, Germany; 5 Department of Urology, HELIOS Hanseklinikum, 18435 Stralsund, Germany

**Keywords:** upper tract urothelial carcinoma, ERBB2, fluorescence in situ hybridization, tissue microarray, survival analysis

## Abstract

**Simple Summary:**

Upper tract urothelial carcinomas (UTUCs) occur in about 5–10% of all urothelial carcinomas and are frequently discovered in high-stage disease. We aimed to evaluate human epidermal growth factor receptor 2 (ERBB2) protein expression and *ERBB2* amplification in UTUCs according to the American Society of Clinical Oncology/College of American Pathologists guidelines for gastric carcinoma, revealing an obvious higher rate of ERBB2 overexpression (41.8%) in contrast to the relatively low frequency of *ERBB2* amplification (10.5%) in UTUCs. Correlation and survival analyses show that ERBB2 overexpression and amplification were linked with high-grade and high-stage tumors and with tumor progression. The results suggest that ERBB2 is a biomarker for progression in UTUCs. As previously shown, *ERBB2* amplification is infrequent. However, the small number of patients diagnosed with *ERBB2*-amplified UTUC might benefit from ERBB2-targeted therapy. For the reliable detection of rare *ERBB2*-amplified UTUC, simultaneous immunohistochemical and cytogenetic ERBB2 analysis is recommended.

**Abstract:**

Upper tract urothelial carcinomas (UTUCs) occur in about 5–10% of all urothelial carcinomas and are frequently discovered in high-stage disease. We aimed to evaluate human epidermal growth factor receptor 2 (ERBB2) protein expression immunohistochemically and *ERBB2* amplification in UTUCs by fluorescence in situ hybridization, applying a tissue microarray technique. ERBB2 overexpression and *ERBB2* amplification were defined according to the recommendations of the American Society of Clinical Oncology/College of American Pathologists (ASCO/CAP) for breast cancer and gastric carcinoma (GC), revealing scores of 2+ and 3+ in 10.2% and 41.8% of UTUCs, respectively. The performance parameters showed obviously higher sensitivity of ERBB2 immunoscoring according to the ASCO/CAP criteria for GC. *ERBB2* amplification was detected in 10.5% of UTUCs. ERBB2 overexpression was more likely to be found in high-grade tumors and was associated with tumor progression. Univariable Cox regression analysis revealed a significantly lower progression-free survival (PFS) in cases with ERBB2 immunoscores of 2+ or 3+ according to the ASCO/CAP guidelines for GC. UTUCs with *ERBB2* amplification showed a significantly shorter PFS in the multivariable Cox regression analysis. Irrespective of their *ERBB2* status, patients with UTUC treated with platin showed a significantly lower PFS than UTUC patients who had not received any platin-based therapy. In addition, UTUC patients with a normal *ERBB2* gene status who had not received platin-based therapy showed significantly longer overall survival. The results suggest that ERBB2 is a biomarker for progression in UTUCs and may define a distinct subgroup of UTUCs. As previously shown, *ERBB2* amplification is infrequent. However, the small number of patients diagnosed with *ERBB2*-amplified UTUC might benefit from ERBB2-targeted cancer therapy. In clinical–pathological routine diagnostics, the determination of *ERBB2* amplification is an established method in some defined entities and also successful in small samples. Still, the simultaneous use of ERBB2 immunohistochemistry and *ERBB2* in situ hybridization would be important in order to record the low rate of amplified UTUC cases as completely as possible.

## 1. Introduction

Primary upper tract (UT) urothelial carcinomas (UC) are, unlike urinary bladder carcinomas (UBCs), a rare subtype of UCs, accounting for only 5–10% of all UCs [1,2]. Upper tract urothelial carcinomas (UTUCs) are frequently discovered in high-stage disease, and more than 40% of diagnosed tumors are T2 or higher at nephroureterectomy [3,4,5]. Altogether, 60% of UTUCs are invasive at diagnosis compared to 15–25% of UBCs [3,5,6]. UTUC is often associated with poor clinical outcome, with a 5-year cancer-specific survival of <50% for T2 or T3 and <10% for T4 tumors [7,8]. The standard approach for localized disease in high-risk patients consists of open radical nephroureterectomy with bladder cuff excision [6,7]. There is a lack of standard treatment options in the adjuvant and neoadjuvant settings [1]. In advanced UTUCs, adjuvant platinum-based chemotherapy is the standard of care after the advent of the POUT study, but due to chemotherapy-related toxicity, particularly nephrotoxicity of platin derivatives, and the risk of an impaired postoperative renal function, not all patients are eligible for platin-based chemotherapy [1,3,9].

The genes of the human epidermal growth factor receptor family (EGFR or ERBB family) encode receptor tyrosine kinases, including EGFR (ERBB1), ERBB2 (also HER2), ERBB3, and ERBB4. *ERBB2* is located on chromosome 17q21 and acts as a type I transmembrane growth factor receptor, which plays a crucial role in cell proliferation and tumorigenesis [10]. *ERBB2* amplification leads to increased levels of ERBB2 at the cell membrane, consecutive homodimerization or heterodimerization with other members of the ERBB family, and results in constitutive self-activation and activation of the phosphoinositide 3-kinase (PI3K)/AKT/mammalian target of rapamycin (mTOR) signaling cascade [11]. The oncogenic potential of *ERBB2* has been well-established in the preclinical and clinical settings. *ERBB2* amplification and protein overexpression play variable roles in diverse cancers such as breast cancer (BC), esophagogastric and gastric cancer (GC), ovarian cancer, UBC, extrahepatic cholangiocarcinoma, lung cancer, and colon cancer [11,12,13]. While current treatment options for advanced UTUCs are limited, the detection of *ERBB2* amplification might provide a rational therapeutic option in these patients, comparable to BC and GC [14,15]. In comparison to UBCs, UTUCs seem to harbor *ERBB2* alterations less frequently, i.e., 8% in UTUCs versus 19% in UBCs [16]. Previously published data have shown different ERBB2 overexpression and *ERBB2* amplification rates in UTUCs, ranging from 8.3% to 74% and from 8% to 18.1%, respectively [17,18,19,20,21,22,23,24,25,26,27]. Elucidating the role of anti-ERBB2 therapy in UBC, Marin et al. stated that the ERBB2 overexpression or *ERBB2* amplification rates vary considerably, in part due to the variability in immunohistochemistry (IHC) assays, cut-off values, antibodies, kits, or protocols applied [28]. Therefore, our working hypothesis is that a standardized evaluation procedure can isolate the patients who are eligible for a ERBB2-specific therapy.

The main research objective was to analyze ERBB2 protein expression comparatively according to the recommendations of the American Society of Clinical Oncology/College of American Pathologists (ASCO/CAP) for ERBB2/HER2 testing in BC [29] and gastroesophageal adenocarcinoma [30] followed by fluorescence in situ hybridization (FISH) for *ERBB2* gain or amplification in UTUCs. The peripheral aim was to investigate the impact of ERBB2 overexpression and *ERBB2* amplification on patient survival.

## 2. Materials and Methods

### 2.1. Study Population and Clinicopathological Data Assessment

This retrospective study included formalin-fixed, paraffin-embedded (FFPE) tissues of a total of 157 patients with 160 primary UT tumors, consisting of 128 infiltrating UCs, 17 non-invasive low-grade papillary UCs, 14 non-invasive high-grade papillary UCs, and one urothelial carcinoma in situ. FFPE samples were retrieved from the archives of the Institute of Pathology at the University Medical Center of Rostock, diagnosed between January 2000 and December 2015. One female patient was diagnosed with a contralateral metachronous tumor of the renal pelvis. In two male patients, separate tumors of the ipsilateral renal pelvis and ureter had been resected. All except three patients had undergone surgery for tumors of the renal pelvis or ureter (nephroureterectomy with or without bladder cuff or ureterectomy) at the Department of Urology at the University Medical Center Rostock between 2000 and 2015. All cases were reclassified according to the updated WHO classification [31,32]. All cases were histologically reviewed according to the growth pattern, grading, TNM classification, lymphovascular invasion, perineural invasion, and the presence of inflammation and necrosis (A.Z.).

Clinical data were collected by reviewing the charts of the Clinical Cancer Registry and the Department of Urology. These data were anonymized and included sex, age at diagnosis, type of surgical intervention, grade, stage, applied chemotherapy regimens and/or radiotherapy, and information concerning progression-free survival (PFS) and overall survival (OS). The patient and tumor characteristics are given in Table 1. In some cases, not all data were available.

The study was performed in accordance with the declaration of Helsinki and German laws concerning data safety, approved by the Ethics Committee of the University of Rostock (reference number: A2016-0015), and with written consent from all patients prior to surgery. Patient data were anonymized according to German laws regulating patient data protection.

### 2.2. Construction of Tissue Microarrays (TMAs)

For TMA construction, a hematoxylin and eosin (H&E)-stained slide from each sample block was used to define representative tumor regions and areas with normal urothelial mucosa. Using a precision instrument (Beecher Instruments, Silver Spring, MD, USA), tissue cylinders (cores) with a diameter of 0.6 or 1.0 mm were punched from the tumor areas of each block and brought into a recipient paraffin block as described previously [33]. Eight different TMA blocks were constructed. To increase the tumor yield and overcome biomarker heterogeneity, each case was punched at least three times, and the corresponding normal urothelium for internal biomarker validation was added [34].

### 2.3. IHC

For evaluation, 1 µm sections of formalin-fixed and paraffin-embedded (FFPE) patient tumors were transferred to microscope slides (DAKO, Hamburg, Germany). Deparaffinization, rehydration, and epitope retrieval were performed at pH = 9 and 95 °C for 20 min according to the manufacturer’s protocol using EnVision™ FLEX (DAKO) in a semi-automated autostainer Link 48 (DAKO). Polyclonal rabbit anti-human primary antibody (DAKO) against c-erbB-2 oncoprotein was diluted 1:6000 in EnVision™ FLEX Antibody Diluent (DAKO). For antigen detection, EnVision™ FLEX+ Rabbit (DAKO) was used.

ERBB2 expression was scored according to the guidelines for ERBB2/HER2 testing in GC and adenocarcinoma of the esophagogastric junction given by the CAP, the American Society for Clinical Pathology (ASCP), and the ASCO, and additionally according to the recently published guidelines for ERBB2/HER2-testing in BC [29,30].

According to the ASCO/CAP 2018 guidelines for ERBB2/HER2 testing in BC, complete negative ERBB2 staining or a faint or barely perceptible incomplete membrane stain in less than 10% of tumor cells was referred to as negative (score 0). An incomplete and faint membrane staining in >10% of tumor cells was scored as 1+. Weak to moderate complete membrane staining in >10% of tumor cells was scored as 2+, and intense complete circumferential membrane staining was categorized as 3+ [29].

In accordance with the ASCO/CAP 2017 guidelines for ERBB2/HER2 testing in GC, no reactivity or membranous reactivity in <10% of tumor cells was referred to as negative (score 0). Faint or barely perceptible membranous reactivity in ≥10% of tumor cells or cells showing a weak reactivity in only a part of their membrane was scored as 1+. Weakly positive to moderate continuous expression through the entire tumor cell membrane or lateral/basolateral membranous reactivity in ≥10% of tumor cells was scored as 2+. Regardless of whether an *ERBB2* amplification or gain might underline the ERBB2 expression or not, a strong continuous ERBB2 expression through the entire cell membrane or lateral/basolateral membranous reactivity in ≥10% of tumor cells was rated as 3+ [30].

### 2.4. FISH

FISH for the detection of *ERBB2* gains or amplifications was conducted on 5 µm-thick histological sections as previously described in detail [35]. The TMA sections were directly labeled with Zyto*Light* SPEC ERBB2/CEN17 dual-color probes (Zytomed Systems, Berlin, Germany). After probing and hybridization, the nuclei were counterstained with DAPI DuraTect Solution (Zytomed Systems). The FISH slides were analyzed and scored by fluorescence microscopy using an Olympus BX53 microscope (Olympus, Hamburg, Germany) equipped with a DP-72 camera (Olympus). Hybridization signals of a minimum of 20 non-overlapping nuclei were manually counted on a single-cell basis (A.Z., M.Mo., B.S.). The ratio was calculated using the total number of *ERBB2* signals divided by the total number of centromere 17 (CEN17) signals. According to the ASCO/CAP 2018 guidelines for *ERBB2* testing in BC [29], *ERBB2* amplification was defined by an *ERBB2*/CEN17 ratio ≥ 2.0 with an average *ERBB2* copy number < or >4.0 signals per cell or an *ERBB2*/CEN17 ratio <2.0 with ≥6.0 signals per cell. The *ERBB2* result was classified as a gain or equivocal if the *ERBB2*/CEN17 ratio was <2.0 and the *ERBB2* copy number was between ≥4.0 and <6.0 signals per cell [29]. The H&E-stained TMA sections were used for reference histology.

### 2.5. Statistical Analysis

Statistical analysis was performed using SPSS 28.0.0.0 software (IBM, Ehningen, Germany). Descriptive statistics were computed for continuous and categorical variables. The statistics computed included the mean and standard deviation (SD) values for the continuous variables, and the frequencies and relative frequencies for the categorical factors. OS and PFS were analyzed using the Kaplan–Meier method. Different groups were compared using the log-rank test. Cox proportional hazards regression (Cox regression) analysis was performed to analyze the covariates with a potential influence on OS and PFS of UTUCs with ERBB2 overexpression (score 2+ or 3+), *ERBB2* amplification, and platin therapy. Multivariable regression models were fitted using complete cases, and imputation procedures for missing values were not carried out. Considering the number of events, backward selection was performed with a maximum of 10 predictors in the final model. To compare differences between the ERBB2 protein expression or *ERBB2* gain or amplification and several clinicopathological variables, Fisher’s exact test or Pearson’s chi-square test was applied. All *p*-values were obtained using two-sided statistical tests, and a *p*-value <0.05 was considered to indicate statistical significance. Determinations of sensitivity, specificity, and positive and negative predictive value were made according to Lenhard and Lenhard [36].

## 3. Results

### 3.1. Tumor and Patients Characteristics

The demographic and clinicopathological data of the 157 patients with UTUC are shown in Table 1. The mean age at diagnosis was 70.14 yrs (range = 34.99–94.19 yrs). Twenty-seven out of one hundred and fifty-seven patients were aged 60 or younger. Survival data (OS and PFS) were available in 138 cases (mean OS = 5.17 yrs, range = 0.005–18.86 yrs) and 123 cases (mean PFS = 5.18 yrs, range = 0.25–18.86 yrs), respectively. Ninety-nine/138 patients died and 30/123 patients experienced disease progression during the observation period. Adjuvant platin-based chemotherapy was started in 24 patients, gemcitabine was administered in 27 patients, and targeted therapy with nivolumab or atezolizumab was started in 2 patients with progressive tumors after platin-based chemotherapy.

Of the 160 tumors included in this study, the following tumor diagnoses were given: 128 (80%) infiltrating UTUCs, 17 (10.6%) non-invasive papillary low-grade UCs, 14 (8.75%) non-invasive high-grade papillary UCs, and 1 (0.6%) urothelial carcinoma in situ. Tumor localization, grading, and TNM staging of the tumors are listed in Table 1. Lymphatic, vascular, and perineural invasion were found in 54, 50, and 19 cases, respectively. Based on previous analyses, the cohort contained 30 highly microsatellite instable (MSI-H) UTUC cases, including 9 MSI-H UTUCs with documented Lynch-associated carcinoma in family history [33].

### 3.2. Morphological Evaluation (H&E Stains)

A total of 155 out of 160 UTUC cases on the TMAs were evaluable and contained >10% tumor tissue. Prior to TMA production, full tissue sections of each UC case were reevaluated with respect to the tumor growth pattern: 49 cases showed an (exophytic) papillary growth, 28 cases a solid tumor growth, 25 cases an inverted papillary or solid-papillary growth pattern, and 52 cases displayed a mixed tumor morphology, mostly with papillary and solid components. Two cases showed micropapillary and three cases sarcomatoid morphology. Tumor necrosis was observed in 63 cases. Major tumor-associated lymphofollicular inflammation was seen in 19 UTUCs.

### 3.3. Determination of ERBB2 Status

#### 3.3.1. ERBB2 Immunoscoring

**Figure 1:**
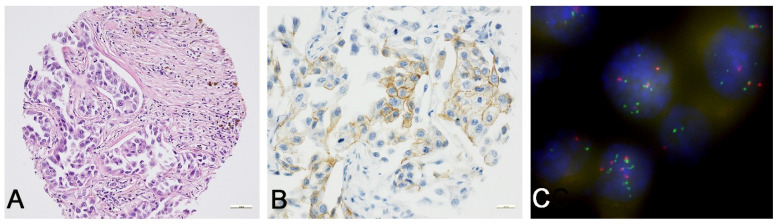
ERBB2 protein expression and *ERBB2* amplification in upper tract urothelial carcinoma. (**A**) Detail of solid and papillary high-grade (G3) UTUC (20×, hematoxylin and eosin). (**B**) In the same case, weak to moderate incomplete ERBB2 staining in >10% of tumor cells was seen (ERBB2/HER2 immunoscore of 2+ in accordance with ASCO/CAP 2017 guidelines for ERBB2/HER2 testing in GC). The heterogeneity of ERBB2 staining is illustrated (ERBB2 immunohistochemistry/DAKO, 40×). (**C**) FISH analysis revealed a low *ERBB2* amplification rate with an *ERBB2/CEN17* ratio of 2.55 and an average *ERBB2* copy number of 6.45 (100×).

According to the recommendations of ERBB2 testing in GC, the scores of 1+, 2+, and 3+ were noted in 17.8%, 29.5%, and 12.3% of UTUCs, respectively (Table 2), mostly with incomplete basolateral membranous ERBB2 staining (Figure 1). Consequently, when compared to the results according to the guidelines of BC (Table 2), 49.3%, 3.4%, and 6.8% of cases displayed scores of 1+, 2+, and 3+, respectively.

#### 3.3.2. ERBB2 FISH Analysis

In 17/160 UTUCs, no signals were detected, and therefore, these cases were not evaluable. In 15/143 cases (10.5%), an *ERBB2* amplification was detected (Table 2), and another 23/143 (16.1%) UTUCs displayed an increase in the *ERBB2* copy numbers. According to the more sensitive ERBB2 immunoscoring scheme for GC, four, five, seven, and six tumors with *ERBB2* gain had ERBB2 scores of 0, 1+, 2+, or 3+, respectively, and zero, one, six, and seven tumors with *ERBB2* amplification displayed ERBB2 scores of 0, 1+, 2+, or 3+, respectively (Figure 1).

#### 3.3.3. Quality Parameters and Correlation Analyses

A comparison of the evaluation schemes with the *ERBB2* amplification status determined by FISH as the gold standard resulted in the following quality features: ERBB2 immunoscoring according to the ASCO/CAP 2018 guidelines for ERBB2/HER2-testing in BC detected 4 true-positive, 111 true-negative, 9 false-positive, and 10 false-negative UTUCs (sensitivity = 30.8%, specificity = 91.7%, positive predictive value = 28.6%, negative predictive value = 92.5%). ERBB2 immunoscoring according to the ASCO/CAP 2017 guidelines for ERBB2/HER2 testing in GC detected 13 true-positive, 77 true-negative, 43 false-positive, and 1 false-negative UTUCs (sensitivity = 92.9%, specificity = 64.2%, positive predictive value = 23.2%, negative predictive value = 98.7%).

ERBB2 overexpression (scores of 2+ and 3+) according to the ASCO/CAP 2018 guidelines in BC was seen in zero, two, and thirteen UTUCs, with WHO grades of 1, 2, or 3, respectively. ERBB2 overexpression was only detected in 15 invasive UTUCs and not in any pTa tumors. An association between positive (score 2+ or 3+) ERBB2 expression according to the ASCO/CAP guidelines in BC and high grade (*p* = 0.012), and between ERBB2 positivity and the presence of an *ERBB2* amplification or gain (*p* < 0.001), but not for growth pattern, necrosis, stage, and other clinicopathological parameters (*p* > 0.05) was noted (Table 3).

ERBB2 overexpression (scores of 2 + and 3+) according to the ASCO/CAP 2017 guidelines in GC was seen in 3, 22, and 36 UTUCs, with WHO grades of 1, 2, or 3, respectively. This difference did not reach the level of significance in Pearson’s chi-square test (*p* = 0.077). Similar to the correlation of expression and tumor grading, ERBB2 overexpression was detected in 39/61 advanced UTUCs staged 2–4, but only in 9 tumors staged 0 and in 13 tumors staged 1 (*p* > 0.359). Further correlation analyses demonstrated significant associations between ERBB2 positivity and tumor progression (*p* = 0.028), and the presence of an *ERBB2* amplification (*p* < 0.001), but not for age, sex, growth pattern, necrosis, or other clinicopathological parameters, such as tumor stage, lymph node metastasis, distant metastasis, and lymphovascular or perineural invasion (*p* > 0.05) (Table 3).

Fisher’s test and Pearson’s chi-square test demonstrated associations between UTUCs with *ERBB2* gain or amplification and grade (*p* < 0.001), invasion (pT1-4 versus pTa, *p* = 0.003), advanced stage (*p =* 0.0014), positive lymph node status (*p* = 0.005), lymphatic invasion (*p* = 0.005), perineural invasion (*p* = 0.005), positive resection margins (residual status; *p* = 0.006), necrosis (*p* = 0.048), and positive ERBB2 immunoreaction, according to both evaluation schemes (both *p* < 0.001). A positive but not significant relationship may have been indicated between UTUCs with *ERBB2* gain or amplification and tumor morphology (*p* = 0.057) and distant metastasis (*p* = 0. 064). UTUCs with *ERBB2* gains or amplification showed a papillary, solid-papillary, solid, or mixed-growth pattern, and only one of two *ERBB2*-amplified cases exhibited a micropapillary pattern. Further statistical analysis, including age, sex, and additional tumor properties revealed no significant differences (*p* > 0.05) (Table 3).

### 3.4. Survival Analysis

**Figure 2:**
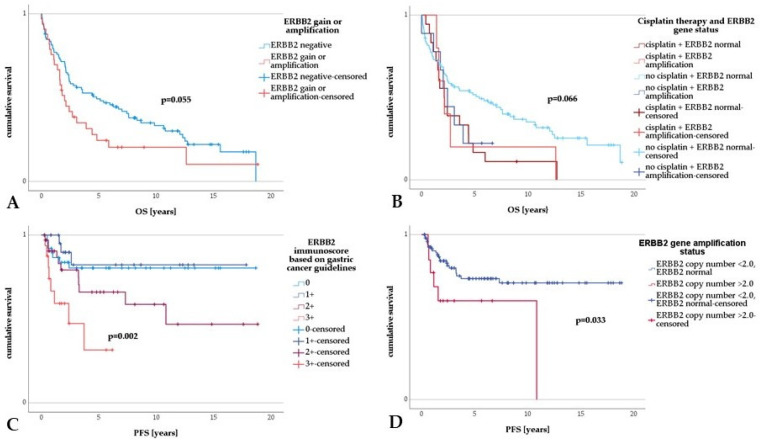
Survival analysis in upper tract urothelial carcinoma. (**A**) Upper tract urothelial carcinoma with *ERBB2* amplification or gain displayed a trend toward shorter OS compared to UTUC without *ERBB2* amplification or gain. (**B**) Comparing different groups, UTUC patients diagnosed with normal *ERBB2* gene status and without adjuvant platin-based treatment showed a significantly longer OS. (**C**) There was a highly significant shorter PFS in UTUCs with ERBB2 immunoscores of 2+ and 3+ in contrast to UTUC with an ERBB2 immunoscore of 0 or 1+. (**D**) A significantly shorter PFS in UTUC with *ERBB2* amplification compared to UTUC with normal *ERBB2* gene status was revealed.

Cox regression analysis illustrated no statistical differences in the OS in cases of ERBB2 protein expression (scores of 2+ or 3+) versus negative ERBB2 protein expression (*p* > 0.05). An overt trend towards shorter OS in UTUCs with *ERBB2* amplification or gain (*p* = 0.057) was seen (Table 4, univariable Cox regression; Figure 2A). A significantly higher OS was noted in UTUC patients with *ERBB2*-normal UTUCs who had not received chemotherapy (*p* = 0.006; Table 4, univariable Cox regression, Figure 2B). Overall, UTUC patients who received platinum-based chemotherapy showed significantly poorer OS, which persisted in multivariable analysis (*p* = 0.005; Table 4 multivariable Cox regression). As for the clinicopathological parameters, G3 tumors showed a lower OS than G1 tumors (*p* = 0.001; Table 4, univariable analysis). In comparison to pTa UTUCs, in high-stage UTUCs (pT3 or pT4), a significantly lower OS was seen (*p* < 0.001; Table 4, univariable analysis). There was also an inferior OS in patients with progressive disease, positive nodal status, distant metastasis, lymphatic or venous invasion, perineural invasion, positive resection margins, necrosis, and sarcomatous growth pattern (*p* = 0.008, *p* < 0.001, *p* < 0.001, *p* < 0.001, *p* < 0.001, *p* < 0.001, *p* < 0.001, *p* = 0.004, *p* < 0.001, respectively; Table 4, univariable analysis).

Concerning PFS, univariable Cox regression analysis revealed a significantly lower PFS in cases with an ERBB2 immunoscore of 2+ or 3+ (*p* = 0.003; Table 5, univariable Cox regression, Figure 2C), a result which did not persist in the multiple regression approach (Table 5, right panel). UTUCs with *ERBB2* amplification showed a significantly shorter PFS (*p* = 0.040; Table 5, univariable Cox regression), a result that was also implied in the multivariable analysis. Irrespective of their *ERBB2* status, patients with UTUC treated with platin showed a significantly lower PFS than patients with UTUC who had not received any platin-based therapy (*p* < 0.001, 95% CI = 3.865–16.851, HR = 8.192; Table 5, univariable Cox regression, Figure 2D), a result which persisted in the multiple regression approach (Table 5, right panel). We re-examined this unexpected result in the number and average age homogenized comparison collectives with similar results. As for the clinicopathological parameters, a lower PFS was seen in tumors with a sarcomatoid growth pattern (*p* = 0.004; Table 5, left panel), in patients with high-stage UTUC (pT4 versus pTa, *p* = 0.006; Table 5, univariable analysis), in patients with lymph node metastasis (*p* = 0.015; Table 5, left panel) or distant metastasis (*p* = 0.001; Table 5, left panel), and in cases with positive resection margins (R1 or R2, *p* = 0.018; Table 5, univariable analysis).

## 4. Discussion

Identifying prognostic and predictive biomarkers in UTUC is crucial for prognostication and therapy decisions for many advanced UTUCs. As an effectual example for urologists, the detection of ERBB2 overexpression and amplification in BC patients is closely related to a poor prognosis, but numerous patients with ERBB2-positive BC in metastatic and adjuvant settings have benefitted from ERBB2-targeted therapy [37,38]. However, unlike in BCs, the prognostic and predictive value of ERBB2 overexpression and *ERBB2* amplification in UTUCs remains uncertain.

In the present study, according to the screening guidelines for GC, a relatively high number of ERBB2-overexpressing UTUCs, with 29.5% scoring 2+ and 12.3% scoring 3+, were detected, in contrast to the relatively low rate of 10.5% of *ERBB2*-amplified UTUCs in the cohort. In particular, these findings agree with the results reported by Aumayer et al., Langner et al., Verhasselt-Crinquette et al., and Yorozu et al., showing ERBB2 overexpression in 16.9–33.3% of UTUCs and *ERBB2* amplification in 8–13.5% of cases [17,22,26,27]. Only the study of Sasaki et al. shows equal frequencies of ERBB2-positive and *ERBB2*-amplified cases of 18.1% each [23]. Established and validated evaluation schemes were used in all of the studies mentioned. Some other studies have been based solely on immunohistochemical data; the frequencies of ERBB2 overexpression have ranged from 8.4–74% [18,19,20,21,24,25]. Except for in the work of Soria et al., specially designed evaluation systems for ERBB2 immunoscoring have been used [18,19,20,21,24,25].

The comparison of the data illustrates the following problems. First, the frequency of ERBB2 overexpression varies widely and reflects the fact that, among other things, different methods, antibodies, and evaluation schemes were used [28]. As for Ménard et al., the literature shows large variation in the ERBB2 levels within one tumor entity, most probably due to the lack of standardized methods for assessing the ERBB2 expression status [11]. Second, according to the guidelines for BC, ERBB2 immunoscoring provides lower frequencies for scores of 2+ and 3+ than the validated scoring system for adenocarcinomas of the stomach or gastroesophageal junction [21]. Similar to the work of Kim et al., the present study illustrates that, as per the recommendations of ERBB2-testing in GC, the frequencies of the scores of 2+ and 3+ are above the ERBB2 values according to the guidelines of BC [21]. The current data show that comparatively few ERBB2 cases scoring 2+ and 3+ (3.4% and 6.8%, respectively) were detected when applying the guidelines for BC. By FISH analysis, 15 (10.5%) UTUCs, with an *ERBB2* copy number of ≥2 were found, which, with the exception of one case, had been detected by the ERBB2 immunoscoring method for GC. ERBB2 immunoscoring according to the guidelines for BC failed to detect 10/15 UTUCs with *ERBB2* amplification. Likewise, Sasaki et al. reported that 38.7% of the *ERBB2*-amplified cases displayed only an ERBB2 immunoscore of 0 or 1+ [23]. Third, evidence of *ERBB2* amplification, which is decisive for a possible therapy option, has only been demonstrated in a small number of UTUCs within a range of 8–18.1% [17,22,26,27]. Thus, it becomes clear that the immunohistochemical overexpression of ERBB2 has reasons additional to the sole amplification of the gene. Furthermore, in other tumor types, the frequency of ERBB2 overexpression has differed in the literature and has often been higher than the gene amplification rate, suggesting that ERBB2 overexpression is due to gene deregulation rather than amplification [11]. This also means that the sole use of immunohistochemistry, even when carrying out validated immunohistochemical tests and evaluation schemes, does not allow for the reliable detection of *ERBB2*-amplified UTUCs for which targeted therapy would be a potential option. In order to identify *ERBB2*-amplified cases as reliably as possible, we propose a simultaneous immunohistochemical and cytogenetic approach: the use of the ASCO/ACP evaluation mode for ERBB2 immunoscoring in GC, since the 2+ UTUC cases in particular often only show incomplete basolateral staining, combined with *ERBB2* FISH or CISH analysis.

ERBB2/HER2 testing guidelines for BC were developed in 2007 by the ASCO/CAP in an attempt to reduce interlaboratory test variability [39]. An algorithm defining positive, equivocal, and negative values for both ERBB2 protein expression and *ERBB2* gene amplification was recommended. A validated surrogate marker for the presence of an *ERBB2* amplification was the strong complete membrane staining of the ERBB2 protein in ≥30% of invasive BC cells. Using an in situ method, the presence of *ERBB2* amplification was defined by an *ERBB2*/CEN17 ratio of >2.2 [39]. Equivocal results in immunohistochemistry (score 2+) as well as in the FISH analysis required additional testing [39]. The ASCO/CAP-recommended ERBB2/HER2 testing criteria for BC was updated, and a threshold of 10% and an *ERBB2*/CEN17 ratio of ≥2.0 were introduced in 2013 to ensure that the appropriate patients received the ERBB2-targeted drug [40]. Additionally, in 2008, ERBB2 testing guidelines in advanced esophago-gastric junction and gastric adenocarcinomas were established to identify suitable patients for trastuzumab therapy [15]. However, validated methods and scoring systems for the determination of *ERBB2* amplification status remain widely unavailable for UTUC. However, despite using an established test system, as for BC or GC, interobserver variability can also contribute to the variability in the ERBB2 status interpretation. This is an important point that must be considered regarding the limitations of our study. The reasons for the variations in ERBB2 overexpression rates are multifactorial. In addition, tumor heterogeneity may play a role as a cause for imprecise ERBB2 analysis. Several factors could lead to the discrepancy of ERBB2 overexpression rates in UTUCs, including technical limitations in immunohistochemistry, the use of subjective scoring systems, and uncertain cut-off-values. Especially in retrospective studies with FFPE material, the problem of tissue preservation with gradual protein degradation is added [41]. Another limitation is the low number of *ERBB2*-amplified UTUCs in this cohort. Despite the limitations of our study, it is obvious that tumors with *ERBB2* amplification or gain represent a subgroup of UTUCs that could probably benefit from ERBB2-targeted therapy. Prospective studies considering this question should follow this research.

According to the EAU Guidelines for UTUC, the tumor stage and grade are the primary recognized prognostic factors [1]. Lymph node metastasis, lymphovascular invasion, extensive necrosis (>10% of the tumor area), and the occurrence of a sessile growth pattern are independent predictors of worse outcomes [1]. In addition, different molecular biomarkers, such as microsatellite instability, E-cadherin, and a high neutrophil-to-lymphocyte ratio have been tested in the past and have shown prognostic impact, but none of these markers have yet met the criteria to support their introduction in daily clinical decision-making [1,42].

A comparison of the two evaluation methods according to the ASCO/CAP guidelines for BC and GC showed that the evaluation scheme according to the guidelines for the GC was significantly more sensitive in the detection of the *ERBB2*-amplified UTUCs. When using the guidelines for BC, 10/14 amplified cases were not recognized. There was no ERBB2 overexpression in non-invasive UCs and a significant relationship to high-grade UTUCs when evaluated according to BC guidelines. Similarly, using the guidelines for GC, ERBB2 overexpression was more likely to be found in high-grade tumors. In particular, in the more sensitive test procedure for GC, the correlation analyses showed associations between ERBB2 overexpression, tumor progression, high tumor grade, and the occurrence of an *ERBB2* amplification or gain. Moreover, in UTUCs with *ERBB2* amplification or gain, significant associations with higher tumor grade, invasion, advanced stage, distant metastasis, positive lymph node status, and positive ERBB2 immunoreaction according to both evaluation schemes were demonstrated. Furthermore, significant relationships were found between UTUCs with *ERBB2* gain or amplification and the presence of lymphovascular invasion, perineural invasion, positive resection margins, and necrosis. With regard to the results of the survival analyses, it should be emphasized in particular that Cox regression analysis revealed a significantly lower PFS in cases with ERBB2 immunoscores of 2+ or 3+ according to the ASCO/CAP guidelines for GC. UTUCs with *ERBB2* amplification showed a significantly shorter PFS in the multivariable Cox regression analysis. Irrespective of their *ERBB2* status, patients with UTUC treated with platin showed a significantly lower PFS than UTUC patients who had not received any platin-based therapy, a result that persisted in the multiple regression approach. Additionally, UTUC patients with a normal *ERBB2* gene status who had not received platin-based therapy showed a significantly longer OS. Compared to recent results, it is indeed unusual that patients treated with platin displayed a significantly shorter PFS than patients with UTUC who had not received any platin-based therapy because the data of the POUT trial clearly showed that disease-free survival was significantly longer in the therapy group [9]. Perhaps there was selection bias in the present retrospective observational study because, possibly, the individuals who received (needed) this therapy had a worse prognosis per se. Additionally, in total, only 24 patients with metastatic tumor disease were shown to have received platinum-containing therapy in the cohort of 160 patients studied. Thus, this is a very small cohort of this monocentric retrospective study that received platinum therapy. This cohort had a lower mean age (66 years versus 70 years) compared to the untreated cohort, so for this reason alone, the two collectives are not homogeneous in comparison. In addition, it is not known whether there were other previous oncologic therapies or recurrences that might have caused a poorer response to the platinum therapy that was given. However, further survival analysis after the homogenization of both collectives showed that the platinum-treated cohort had a highly significant shorter PFS compared to the untreated group, so the data appear valid despite the small case number. This result would definitely need to be validated in a larger multicenter and prospective study. In the literature, several studies have shown that ERBB2 overexpression and *ERBB2* amplification are significantly associated with features of biologically aggressive tumors and poor prognoses. Similarly to the present study, Soria et al. demonstrated that ERBB2 overexpression was associated with pathologic characteristics, such as a more advanced T stage, high-grade tumors, and the presence of lymph node metastasis, lymphovascular invasion, and tumor necrosis [24]. Furthermore, it was shown that ERBB2 overexpression is a marker for the increased risk of disease progression [24]. In addition, compared to the results presented, patients with ERBB2 overexpression had an increased risk of death, particularly of cancer-specific death [24]. Aumayr et al. were able to show in a similar way that ERBB2 overexpression in UTUC was associated with higher-grade tumors, non-organ-confined carcinomas, and *ERBB2* amplification [17]. The occurrence of *ERBB2* amplification was also associated with a higher tumor grade and lymph node metastasis [17]. According to the data presented, Galanakis et al. and Imai et al. have also shown that ERBB2 overexpression is associated with a significantly shorter time interval to recurrence [19,20]. Tsai et al. showed that the incidence of subsequent tumor recurrence in the urinary bladder correlated significantly with ureteral tumor involvement and ERBB2 expression [25]. In addition, Sasaki et al. reported that *ERBB2* amplification in UTUC was significantly associated with shorter recurrence time in the urinary bladder after nephroureterectomy [23]. Verhasselt-Crinquette et al. reported a significant association only with the nodal stage but not with outcomes [26]. Further studies have also shown that both ERBB2 overexpression and the occurrence of *ERBB2* amplification were linked to UTUCs with adverse biological characteristics and an adverse outcome [18,21,22,27]. These results imply that ERBB2-overexpressing or *ERBB2*-amplified UTUCs represent a distinct molecular subset. Previous attempts have been made to establish a molecular classification of UCs, and a consensus classification for muscle-invasive UBC has recently been published [43]. The majority of *ERBB2*-amplified cases are found in the group of so-called “luminal unstable” UCs. For UTUCs, there are still few data available.

Based on the POUT trial, adjuvant platin therapy within 90 days after nephroureterectomy was recently included in the guidelines for the treatment of UTUC in Germany [9]. Further, due to the lack of relevant therapy studies, targeted therapy has not yet been considered in the standard of care guidelines for UTUC patients [1]. However, only a few patients are eligible for platin after radical nephroureterectomy, mainly due to decreased renal function [44]. For these reasons, new, effective, and less-toxic therapies are needed. Targeted therapies against ERBB2 are currently used in the treatments of BC and GC and could also represent a new and effective option for UTUCs. Previous therapy studies with the ERBB2-specific antibody trastuzumab (Herceptin^®^) in the UC of the bladder did not provide satisfactory results, probably because of insufficient patient selection and the low frequency of UC carrying an *ERBB2* amplification [45]. Similar to bladder UC, the frequency of *ERBB2* amplifications in UTUCs is low, and further prospective therapy studies need to address this issue. This also means careful patient selection and the use of simultaneous ERBB2 testing according to validated guidelines using both immunoscoring and in situ hybridization.

## 5. Conclusions

The present study shows relatively high levels of ERBB2 expression, consistent with the literature, but only a minority of UTUCs harbor *ERBB2* amplification. However, these cases are usually aggressive tumors that are unlikely to benefit from standard adjuvant chemotherapy. The discrepancy between immunohistochemical and cytogenetic findings must be taken into account by a simultaneous procedure using a structured evaluation mode of immunohistochemistry combined with FISH or CISH analysis. With this procedure, smaller biopsies from the upper tract can also be examined so that the basis for a possible individual adjuvant therapy decision could be laid and a possible targeted therapy option could be offered to patients with advanced tumors.

## Figures and Tables

**Table 1:** Patient and tumor characteristics.

Number of Patients		157
Age and gender n (%)		Mean 70.14 yrs, range 34.99–94.19 yrs 99 (63.1) males, 58 females (36.9)
Localization n (%) Total n = 160	Renal pelvis Ureter Renal pelvis and ureter	99 (62.7) 34 (21.5) 25 (15.8)
WHO grading n (%) Total n = 160	G1 G2 G3	17 (10.6) 60 (37.5) 83 (51.9)
Tumor size (mm)	Mean ± SD Range	44.23 ± 32.93 1–230
pT n (%) Total n = 160	a 1 2 3 4 is	31 (19.4) 36 (22.5) 17 (10.6) 50 (31.3) 25 (15.6) 1 (0.6)
pN n (%) Total n = 114	0 1 2	82 (71.9) 6 (5.3) 26 (22.8)
cM n (%) Total n = 106	0 1	91 (85.8) 15 (14.2)
L n (%) Total n = 141	0 1	87 (61.7) 54 (38.3)
V n (%) Total n = 142	0 1 2	92 (64.8) 48 (33.8) 2 (1.4)
Pn n (%) Total n = 69	0 1	50 (72.5) 19 (27.5)
Residual status n (%) Total n = 152	0 1 2	119 (78.3) 23 (15.1) 10 (6.6) *
Growth pattern n (%) Total n = 160	Papillary Papillary-solid/inverted Solid Mixed pattern Micropapillary Sarcomatoid Cis	49 (30.6) 25 (15.6) 28 (17.5) 52 (32.5) 2 (1.3) 3 (1.9) 1 (0.6)
Necrosis n (%) Total n = 128	Absent Present (>10%)	65 (50.8) 63 (49.2)
Chronic lymphofollicular inflammation n (%) Total n = 128	Absent Present	115 (85.8) 19 (14.2)

Abbreviations: Cis—carcinoma in situ, SD—standard deviation, yrs—years. * Note: R2 status included cases with complete tumor resection but presence of distant metastasis at time of diagnosis.

**Table 2:** Immunoscoring of ERBB2 and ERBB2 gain or amplification in upper tract urothelial carcinoma.

ERBB2 Immunoscore	Immunoscoring (According to ASCO/CAP 2018 Guidelines for ERBB2/HER2 Testing in BC)	Immunoscoring (According to ASCO/CAP 2017 Guidelines for ERBB2/HER2 Testing in GC)	Number of Amplified *ERBB2* Cases
	n (%)	n (%)	n (%)
0	59 (36.9)	59 (36.9)	0
1+	72 (45.0)	26 (16.3)	1 (6.7)
2+	5 (3.1)	43 (26.9)	6 (40.0)
3+	10 (6.3)	18 (11.3)	7 (46.7)
n.a.	14 (8.8)	14 (8.8)	1 (6.7)
Total n	160 (100)	160 (100)	15 (100)

**Table 3:** Associations between ERBB2 expression, *ERBB2* gene status, and different clinicopathologcal parameters.

Patients and Tumor Properties	ERBB2-Immunoscoring According to Guidelines for Breast Cancer	ERBB2-Immunoscoring According to Guidelines for Gastric Adenocarcinoma	*ERBB2* Amplification or Gain
	*p*-Value	*p*-Value	*p*-Value
Gender (male, female)	0.994	0.537	0.557
Age ≤ 60 yrs, >60 yrs	0.041	0.820	1.000
Survival	0.939	0.906	0.939
Progression	0.195	0.028	0.549
Morphology (papillary, papillary-solid/inverted, solid, mixed pattern, micropapillary, sarcomatoid, cis)	0.945	0.805	0.057
Necrosis	0.204	0.146	0.048
Lymphofollicular inflammation	0.578	0.680	0.356
WHO grading (G1, G2, G3)	0.012	0.077	<0.001
Invasion, pTa or T1–4	0.257	0.359	0.003
Stage	0.701	0.226	0.014
pN	0.180	0.145	0.005
cM	0.547	0.878	0.064
L	0.496	0.676	0.005
V	0.893	0.389	0.065
Pn	0.773	0.862	0.005
Residual status	0.682	0.883	0.006
*ERBB2* amplification or gain	<0.001	<0.001	-

Statistical tests applied: Pearson chi-square test, Fisher’s exact test.

**Table 4:** Univariable and multivariable Cox regression analysis: overall survival in upper urinary tract urothelial carcinoma.

Overall Survival	Univariable Analyses			Multivariable Analysis		
Parameter	*p*-Value	95% CI	HR	*p*-Value	95% CI	Adj HR
Age > 60 yrs vs. * ≤ 60 yrs	0.081	0.940–2.925	1.658	-	-	-
Sex m vs. * f	0.615	0.736–1.678	1.112	-	-	-
Progression vs. * no progression	0.008	1.178–3.001	1.880	0.451	0.324–12.674	2.025
Pelvic localization vs. ureter vs. * both	0.711	0.818–1.344	1.048	-	-	-
Growth patterns: * Papillary vs. papillary-inverted/solid * Papillary vs. solid * Papillary vs. mixed type * Papillary vs. micropapillary * Papillary vs. sarcomatoid	0.007 0.961 0.010 0.160 0.077 <0.001	0.504–1.919 1.214–4.066 0.864–2.432 0.868–15.725 2.899–35.123	0.983 2.222 1.449 3.694 10.09	0.002 0.049 0.763 0.209 0.579 <0.001	0.032–0.990 0.175–10.757 0.556–14.580 0.078–109.66 7.614–1364.9	0.178 1.373 2.846 2.826 101.942
Necrosis vs. * no necrosis	0.004	1.230–3.046	1.936	0.149	0.752–6.505	2.212
Chronic lymphofollicular inflammation vs. * no inflammation	0.983	0.558–1.771	0.994	-	-	-
ERBB2 score ** 0 vs. 1+ vs. 2+ vs. * 3+	0.193	0.937–1.380	1.137	-	-	-
*ERBB2* amplification vs. * *ERBB2* <2.0	0.176	0.827–2.822	1.528	-	-	
*ERBB2* gain or amplification vs. * no *ERBB2* gain or amplification	0.057	0.986–2.485	1.565	0.114	0.623–83.766	7.222
Platin vs. * no platin-based therapy	0.005	1.243–3.316	2.031	0.043	0.003–0.914	0.049
*ERBB2* status and CT	0.006			0.020		
*ERBB2* negative and CT vs. * *ERBB2* negative and no CT	0.009	1.227–4.223	2.276	0.003	0.009–0.377	0.059
ERBB2 positive and CT vs. * *ERBB2* negative and no CT	0.620	0.504–3.160	1.262	0.021	0.003–0.624	0.046
ERBB2 positive and no CT vs. * *ERBB2* negative and no CT	0.003	1.341–4.028	2.324	0.086	0.003–1.458	0.071
Grading	<0.001			0.071		
G2 vs. * G1	0.125	0.828–4.709	1.974	0.738	0.169–3.522	0.772
G3 vs. * G1	0.001	1.692–9.185	3.942	0.027	0.001–0.663	0.026
pT	<0.001			0.004		
pT1 vs. * pTa	0.060	0.970–4.310	2.045	0.003	2.409–68.305	12.827
pT2 vs. * pTa	0.040	1.044–5.859	2.474	0.019	1.711–415-792	26.674
T3 vs. * pTa	<0.001	1.673–6.410	3.274	0.012	2.346–1021.94	48.959
pT4 vs. * pTa	<0.001	5.006–23.279	10.795	<0.001	11.761–8238.9	311.28
pN1 or pN2 vs. * pN0	<0.001	1.921–5.125	3.138	0.678	1.189–4.677	2.358
cM10 vs. * cM0	<0.001	2.910–10.993	5.656	0.384	0.346–15.830	2.340
Lymphatic invasion (L1 vs. * L0)	<0.001	1.525–3.602	2.344	<0.001	4.130–71.603	17.196
Venous invasion (V1 or V2 vs. * V0)	<0.001	1.483–3.465	2.267	0.090	0.095–1.187	0.335
Perineural invasion (Pn1 vs. * Pn0)	<0.001	1.105–12.198	3.6718	0.442	0.229–1.903	0.660
Residual status (R1 or R2 vs. * R0)	<0.001	2.917–7.280	4.608	<0.001	4.116–76.914	17.793

Abbreviations: Adj—adjusted, CI—confidence interval, CT—platin-based polychemotherapy, HR—hazard ratio, OS—overall survival, vs.—versus; Notes: * reference category, ** ERBB2 immunoscoring scheme for gastric adenocarcinoma [30]. To select OS influencing factors for the multiple regression approach, the cut-off was set as *p* = 0.060.

**Table 5:** Univariable and multivariable Cox regression analysis: progression-free survival in upper urinary tract urothelial carcinoma.

Progression-Free Survival	Univariable Analyses			Multivariable Analysis		
Parameter	*p*-Value	95% CI	HR	*p*-Value	95% CI	Adj HR
Age > 60 yrs vs. ≤ * 60 yrs	0.909	0.430–2.579	1.054	-	-	-
sex m vs. * f	0.609	0.384–1.754	0.820	-	-	-
Pelvic localization vs. ureter vs. * both	0.181	0.872–2.067	1.342	-	-	-
Growth patterns: * Papillary vs. papillary-inverted/solid * Papillary vs. solid * Papillary vs. mixed type * Papillary vs. micropapillary * Papillary vs. sarcomatoid	0.019 0.333 0.944 0.070 0.983 0.004	0.099–2.193 0.277–3.963 0.938–5.135 0.000 2.799–246.9	0.465 1.048 2.195 0.000 26.29	0.869	0.583–1.577	0.959
Necrosis vs. * no necrosis	0.127	0.852–3.620	1.756	-	-	-
Chronic lymphofollicular inflammation vs. * no inflammation	0.663	0.275–2.274	0.791	-	-	-
ERBB2 score ** 1+ vs. 2+ vs. * 3+ vs. * 0	0.003	1.2132.517	1.748	0.176	0.839–2.613	1.480
*ERBB2* amplification vs. * no amplification	0.040	1.042–6.445	2.592	0.032	1.112–11.089	3.511
Platin vs. * no platin-based therapy	<0.001	3.865–16.851	8.192	<0.001	4.596–45.811	14.510
*ERBB2* status and CT	<0.001			0.296	0.419–1.303	0.739
*ERBB2* negative and CT vs. * *ERBB2* negative and no CT	0.859	0.271–2.968	0.898			
ERBB2 positive and CT vs. * *ERBB2* negative and no CT	0.014	0.052–0.717	0.194			
ERBB2 positive and no CT vs. * *ERBB2* negative and no CT	<0.001	0.056–0.338	0.137			
Grading	0.154			-	-	-
G2 vs. * G1	0.450	0.396–8.083	1.789			
G3 vs. * G1	0.125	0.727–13.78	3.166			
pT	0.037			0.791	0.546–1.587	0.930
pT1 vs. * pTa	0.665	0.359–4.988	1.337			
pT2 vs. * pTa	0.491	0.378–7.611	1.696			
pT3 vs. * pTa	0.059	0.958–9.096	2.951			
pT4 vs. * pTa	0.006	1.690–24.657	6.456			
pN1 or pN2 vs. * pN0	0.015	1.234–7.247	2.991	0.070	0.082–1.101	0.300
cM10 vs. * cM0	0.001	2.274–28.137	8.000	0.147	0.629–22.304	3.745
Lymphatic invasion (L1 vs. * L0)	0.121	0.852–3.975	1.840	-	-	
Venous invasion (V1 or V2 vs. * V0)	0.172	0.790–3.738	1.718	-	-	-
Perineural invasion (Pn1 vs. * Pn0)	0.288	0.538–8.083	2.085	-	-	-
Residual status (R1 or R2 vs. * R0)	0.018	1.201–6.833	2.865	0.581	0.139–3.023	0.648

Abbreviations: Adj—adjusted, CI—confidence interval, CT—platin-based polychemotherapy, HR—hazard ratio, OS—overall survival, vs.—versus; Notes: * reference category, ** ERBB2 immunoscoring scheme for gastric adenocarcinoma [30]. To select OS influencing factors for the multiple regression approach, the cut-off was set as *p* = 0.050.

## Data Availability

The original data will be provided upon request.
